# New Phylogenetic Grouping of Positive-Sense RNA Viruses Is Concordant with Replication Complex Morphology

**DOI:** 10.1128/mBio.01402-19

**Published:** 2019-07-30

**Authors:** Tero Ahola

**Affiliations:** a Department of Microbiology, Faculty of Agriculture and Forestry, University of Helsinki, Helsinki, Finland; Columbia University College of Physicians & Surgeons

## LETTER

The idea that positive-sense RNA (+RNA) viruses may be to some extent taxonomically unified arose when unexpected similarities between animal and plant virus genomes were discovered (reviewed in reference 1). A comprehensive early analysis proposed three supergroups (2), which were named alpha-like, flavi-like, and picorna-like. However, the positions of several virus groups (e.g., nodaviruses, tombusviruses, nidoviruses, and leviviruses) have remained uncertain and varied in different analyses. Recent metagenomic studies have revealed additional, yet-uncharacterized virus groups and increased the amount of data (3), permitting a more powerful analysis based exclusively on RNA-dependent RNA polymerase (RdRp) sequences (4). Wolf et al. (4) divide +RNA viruses into three phylogenetic groups in a novel way. Relatively small “branch 1” contains +RNA bacteriophages (i.e., leviviruses) and their eukaryote-infecting relatives, which have soluble polymerases (5). In contrast, the vast majority of animal and plant +RNA viruses replicate in association with various intracellular membranes (6) and were placed onto branches 2 and 3. Branch 2 contains the well-studied picorna-like viruses, as well as nidoviruses. Their replication complexes display a complex morphology of vesicle clusters and double-membrane vesicles, whose detailed organization is poorly understood (7). Branch 3 represents a new phylogenetic cluster, as it unifies the alpha-like and flavi-like viruses as well as nodaviruses and tombusviruses. All these induce the production of spherules, small membrane invaginations, which are proposed to protect the double-stranded replication intermediate and release the completed positive-sense strands through a narrow neck structure (6, 8).

The question thus arises, whether the membrane association of the polymerase was a primordial characteristic of viruses in branches 2 and 3, which may have facilitated virus replication in early eukaryotic cells possessing internal membranes. The phylogeny of RdRps cannot answer this question, since the membrane-binding sites in viral replicases reside outside the conserved core RdRp domain (6). Furthermore, the membrane association mechanisms are entirely different in different virus groups, consisting, e.g., of an amphipathic alpha helix (binding membranes in a monotopic fashion) in several alpha-like viruses, versus multiple transmembrane proteins in flavi-like viruses (7, 9). The second question, therefore, is whether the two types of morphologies of the replication sites for viruses within branches 2 and 3 are ancient features with at least some degree of functional or structural conservation or whether the similar appearances of the replication structures merely reflect our limited understanding. Structurally, the best model of a spherule (from a nodavirus) shows a multimeric polymerase complex forming a ring or crown at the neck of the spherule (10). The conservation of such an arrangement, even if it consists of a different set of proteins in a different stoichiometry, may reflect a common ancestry, if an evolutionary pathway connecting the different structures can be reconstructed. However, complex structures can also arise through convergence in response to evolutionary pressures, such as the need to protect the replicative RNA. While criticism can be aimed toward a phylogenetic proposal based on a single protein, I believe that the proposed classification of Wolf et al. (4) provides new research directions and increased understanding when complemented with the characterization of newly identified virus groups and with more precise functional and structural models of +RNA virus replication complexes.
